# Ciliated (FOXJ1^+^) Cells Display Reduced Ferritin Light Chain in the Airways of Idiopathic Pulmonary Fibrosis Patients

**DOI:** 10.3390/cells11061031

**Published:** 2022-03-18

**Authors:** Sofia C. Wijk, Pavan Prabhala, Anna Löfdahl, Annika Nybom, Stefan Lang, Hans Brunnström, Leif Bjermer, Gunilla Westergren-Thorsson, Mattias Magnusson

**Affiliations:** 1 Division of Molecular Medicine and Gene Therapy, Lund Stem Cell Center, Lund University, 223 62 Lund, Sweden; sofia.wijk@med.lu.se (S.C.W.); pavan.prabhala@med.lu.se (P.P.); 2 Division of Lung Biology, Department of Experimental Medical Science, Lund University, 223 62 Lund, Sweden; anna.lofdahl@med.lu.se (A.L.); annika.nybom@med.lu.se (A.N.); gunilla.westergren-thorsson@med.lu.se (G.W.-T.); 3 Division of Molecular Hematology, Lund Stem Cell Center, Lund University, 223 62 Lund, Sweden; stefan.lang@med.lu.se; 4 Department of Clinical Sciences, Lund University, 223 62 Lund, Sweden; hans.brunnstrom@med.lu.se; 5 Division of Respiratory Medicine and Allergology, Department of Clinical Sciences, Lund University, 223 62 Lund, Sweden; leif.bjermer@med.lu.se

**Keywords:** IPF, ciliated cells, basal cells, single-cell RNA sequencing, immunofluorescence, ferritin light chain, stem cell therapy

## Abstract

Cell-based therapies hold great promise in re-establishing organ function for many diseases, including untreatable lung diseases such as idiopathic pulmonary fibrosis (IPF). However, many hurdles still remain, in part due to our lack of knowledge about the disease-driving mechanisms that may affect the cellular niche and thereby possibly hinder the function of any transplanted cells by imposing the disease phenotype onto the newly generated progeny. Recent findings have demonstrated increased ciliation of lung cells from IPF patients, but how this affects ciliated cell function and the airway milieu is not well-known. Here, we performed single-cell RNA sequencing on primary ciliated (FOXJ1^+^) cells isolated from IPF patients and from healthy control donors. The sequencing identified multiple biological processes, such as cilium morphogenesis and cell signaling, that were significantly changed between IPF and healthy ciliated cells. Ferritin light chain (FTL) was downregulated in IPF, which suggests that iron metabolism may be affected in the IPF ciliated cells. The RNA expression was confirmed at the protein level with histological localization in lung tissue, prompting future functional assays to reveal the potential role of FTL. Taken together, our data demonstrate the importance of careful analyses in pure cell populations to better understand the IPF disease mechanism.

## 1. Introduction

Idiopathic pulmonary fibrosis (IPF) is a chronic lung disease with severe symptoms and a low survival rate with a median survival after diagnosis of 2–3 years [1]. IPF is the most common type of interstitial lung disease (ILD), affecting about 50 people in 100,000 [2]. Though its incidence is low compared to other chronic inflammatory disorders, such as asthma and chronic obstructive pulmonary disease (COPD), there are currently few effective treatments and no cure except for lung transplantation, which is difficult for patients to access. In addition, the highest risk factor for developing IPF is age, with a major increase in incidence among persons above the age of 75 [3], and with the global population getting older [4], curing age-related diseases such as IPF becomes increasingly important.

IPF has previously been reported to primarily affect lung tissue between alveoli, with changes in the extracellular matrix and fibrosis [3]. However, since bronchiolization of the distal lungs and honeycomb cysts are common features in IPF [5,6], increased attention has been given to the contribution of the airway epithelium in IPF pathogenesis [7].

The majority of the nonalveolar airway epithelium consists of ciliated cells, which function as the first line of defense against inhaled pathogens [8]. In addition, the mucociliary clearance function provided by the ciliated epithelium may also transport small signaling molecules present in the airway microenvironment, contributing to signaling between cells for immune cell recruitment or fibroblast migration, which could possibly trigger or exacerbate pathogenesis [9]. Upon injury or infection, the ciliated cells are replenished by the basal stem cells in the airways [10]. However, not much is known about the role of ciliated epithelial cells in IPF and how they may contribute to the disease and possibly influence the underlying basal stem cells. Examining their potential role is important in order to explore future treatments based on stem cell therapy.

Thus, this study aims to investigate the potential role of ciliated epithelial cells in IPF in order to understand disease-related changes and potentially illuminate new cell-based approaches to treat IPF.

## 2. Materials and Methods

### 2.1. Patient Samples

Human lung tissue (fresh tissue from healthy donors and fresh tissue from explants from IPF) was obtained from Lund University Hospital and Sahlgrenska University Hospital. All donors gave informed consent, and ethical approval for the study was obtained from the Swedish Ethical Review Authority (ethical permit number 1026-15 and ethical permit number 2008-413). For patient information, see Supplementary Table S1.

### 2.2. Tissue Dissociation

Single-cell suspensions were prepared from distal lung tissue pieces and were dissociated according to previously established protocol [11]. Single-cell suspensions were frozen and stored at −150 °C until subsequent thawing for use in experiments.

### 2.3. Fluorescence-Activated Cell Sorting (FACS)

Cells were thawed and resuspended in staining buffer (PBS with 2%FBS) and incubated with fluorescent antibodies (see Supplementary Table S2) and analyzed using a FACS Aria III. After debris, doublets and dead cells were excluded, and epithelial cell enrichment was performed by sorting CD45-CD31-cells for subsequent RNA sequencing.

### 2.4. Single-Cell RNA Sequencing

Single-cell RNA sequencing was performed using the 10x genomics system, and the data were processed as reported previously [11]. All data are in the process of being uploaded to the National Center for Biotechnology Information (Gene Expression Omnibus).

### 2.5. Immunohistochemistry

Immunohistochemistry was performed on formalin-fixed, paraffin-embedded distal lung tissue sections (4 µm thick). After rehydration with xylene and ethanol according to standard procedure, HIER with pH 6.1 was used with DAKO PT link (Dako, Agilent Technologies, Santa Clara, CA, USA). Immunofluorescent staining was performed as reported previously [11]. For antibodies used, see Supplementary Table S3. For negative controls, only secondary antibodies were added. Adjacent tissue sections were stained with hematoxylin–eosin according to standard procedure. Images were obtained with a VS120 virtual fluorescence microscopy slide scanning system (Olympus, Tokyo, Japan), with 20× magnification. From the scanned slides, representative images were acquired using the OlyVIA software V3.2.1 (Olympus, Tokyo, Japan). Qupath [12] was used to quantify ciliated cells (FOXJ1^+^) and FTL+cells in a number of randomly chosen nonbronchial airways from healthy and IPF patient tissue sections (IPF *n* = 4 individuals, 14 airways; healthy *n* = 4, 11 airways). The FTL expression was calculated as the percentage of FOXJ1^+^ cells that expressed FTL.

## 3. Results

### 3.1. Immunohistochemistry and Immunofluorescence Show Typical Structures Associated with Disease in Lung Tissue from IPF Patients

**Figure 1:**
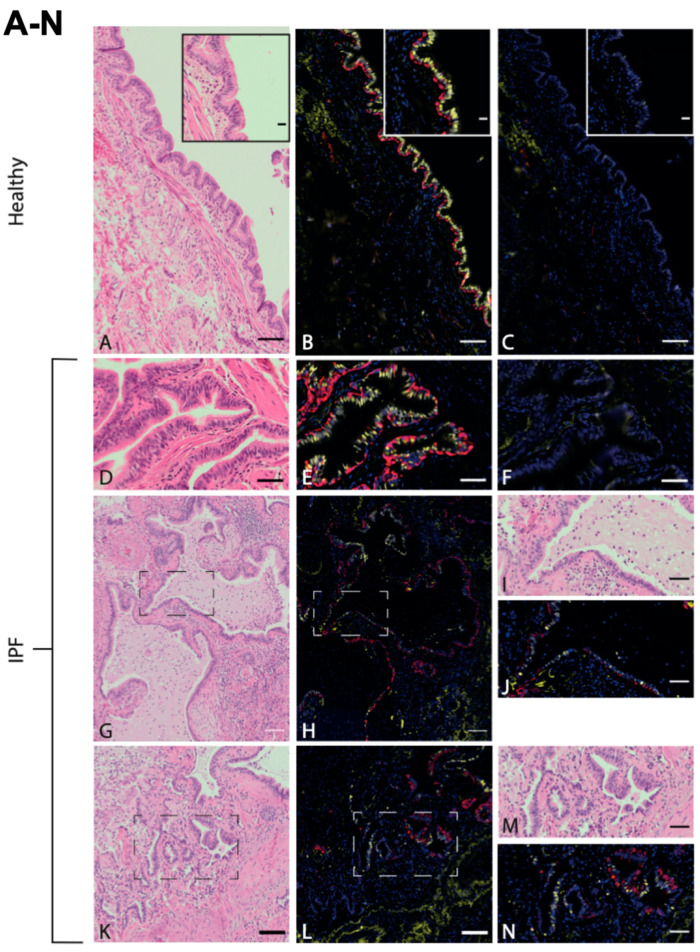
Ciliated airway epithelium in distal lung tissue in healthy and IPF. Antibody labeling of basal cells (KRT5, red) and ciliated cells (FOXJ1, yellow) in airway epithelium of distal lung tissue derived from healthy individuals ((**A**–**C**), enclosed within an enlarged area of ciliated epithelium of terminal bronchiole), and IPF patients (**D**–**F**), all with corresponding H&E stains (**A**,**D**) and negative controls (**C**,**F**). Honeycomb cysts, positive for FOXJ1 and KRT5 in IPF (**G**,**H**), with enlarged visualization of marked area (**I**,**J**). Pulmonary region of suspected bronchiolization (**K**,**L**) with enlarged visualization of marked area (**M**,**N**). Images are representative of examined lung tissue samples (IPF *n* = 4 individuals, healthy *n* = 3). Scale bar: 100 μm (**A**–**C**,**G**,**H**,**K**,**L**), 50 μm (**D**–**F**,**I**,**J**,**M**,**N**), 20 μm (enlargement in (**A**–**C**)).

In order to first visualize the epithelial structures of the sequenced lung tissue, lung tissue sections from healthy donors and IPF patients were stained with hematoxylin–eosin and with KRT5 (basal cells) as well as FOXJ1 (ciliated cells). Representative images of the airway epithelium lined apically with FOXJ1^+^ cells and basally with KRT5^+^ cells from healthy and IPF samples can be seen in Figure 1. Figure 1A,B show a healthy epithelium in terminal bronchioles consisting of basal cells (KRT5^+^, red) and ciliated cells (FOXJ1^+^, yellow) in a pseudostratified layer. Similar epithelial layers were identified in the IPF samples, together with typical pathological features of IPF such as honeycomb cysts. These were lined with epithelial cells positively stained for FOXJ1 and KRT5 (Figure 1G–I). This staining pattern was also observed in pulmonary areas of suspected bronchiolization (Figure 1K–N). The presence of disease-typical structures in the studied IPF lung tissue suggests that the RNA sequencing analysis should mirror important disease mechanisms.

### 3.2. Single-Cell RNA Sequencing Identifies Molecular Differences between Ciliated Cells from IPF Patients and Healthy Control Cells

**Figure 2:**
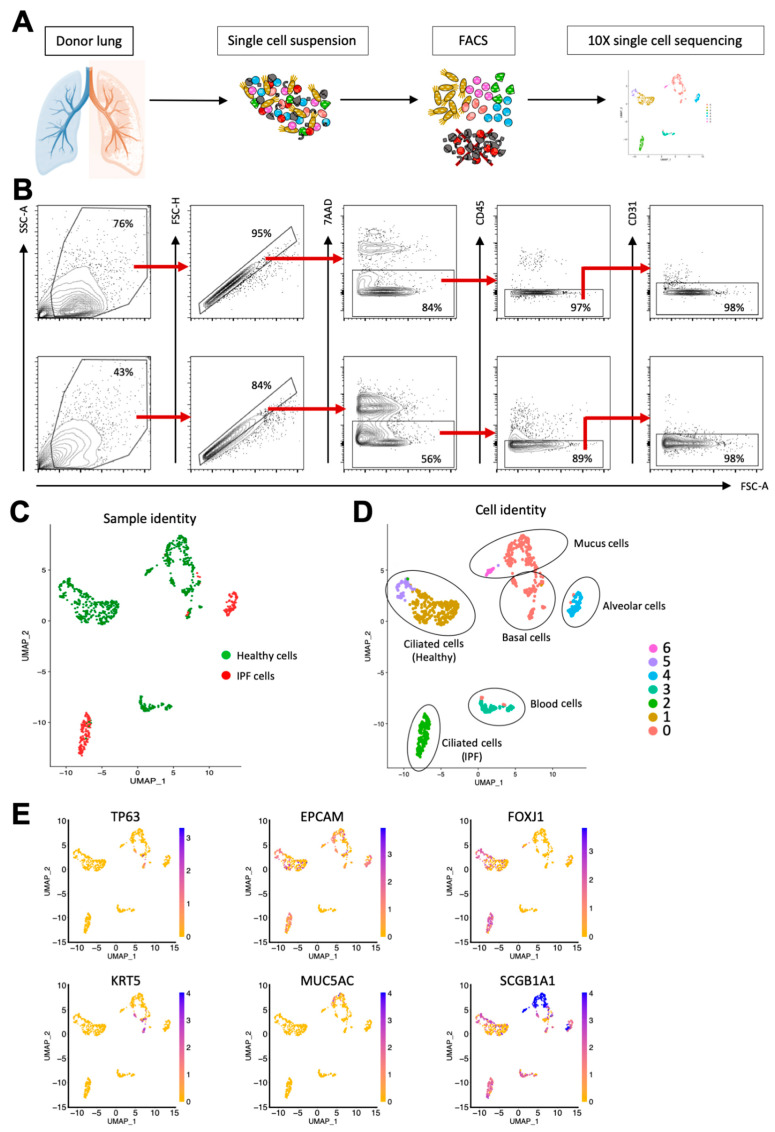
Single-cell sequencing of healthy and IPF lung epithelium identifies common epithelial cell types as well as gene expression differences between healthy and diseased cells. (**A**) Workflow from tissue dissociation to single-cell sequencing. (**B**) Epithelial enrichment by FACS. Top row: Healthy lung tissue. Bottom row: IPF lung tissue. (**C**) UMAP plot showing sample identity of sequenced cells. (**D**) UMAP plot showing sequenced cells clustered according to gene expression. (**E**) UMAP plots showing the expression of known epithelial cell markers.

In order to investigate the potential role of ciliated airway cells in IPF pathogenesis, we obtained lung tissue from two IPF patients as well as four healthy controls. To enrich ciliated cells, we sorted all 7AAD-CD45-CD31-cells using flow cytometry after tissue dissociation (Figure 2A). Figure 2B shows the FACS profiles and gating strategy used for epithelial cell enrichment. Next, to identify molecular changes, we performed single-cell sequencing using the 10x platform according to our previously established protocol [11] (Figure 2A).

**Figure 3:**
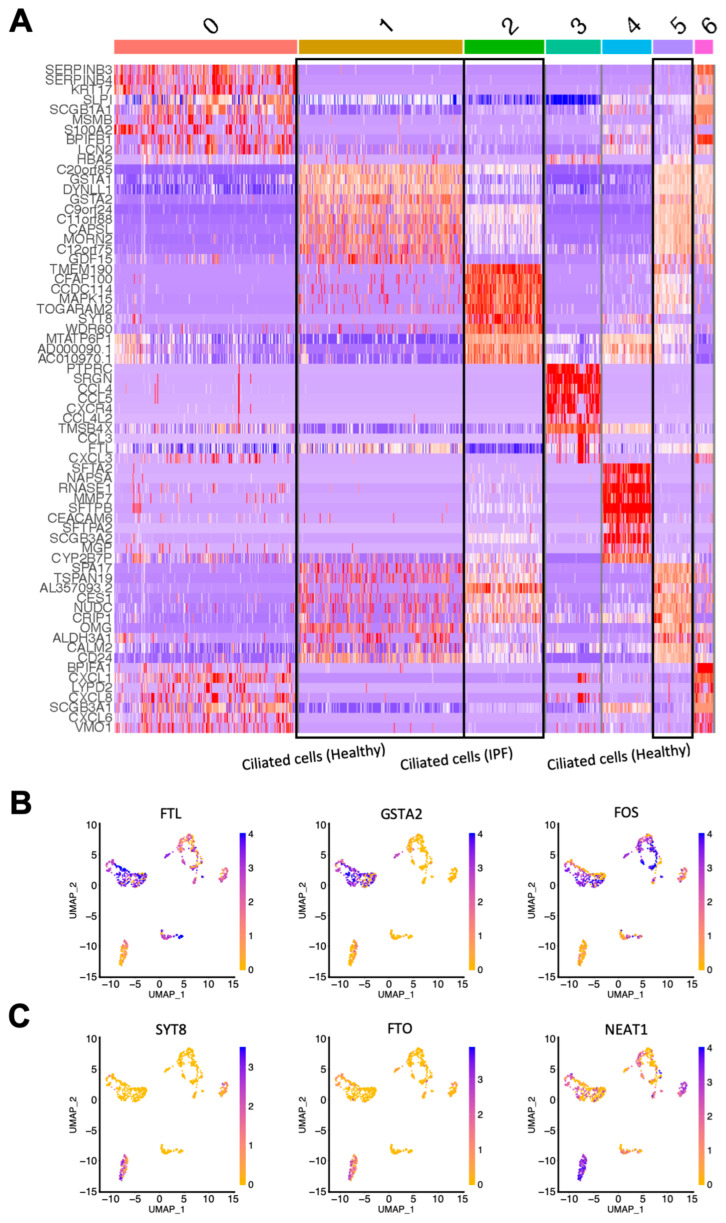
Single-cell gene expression reveals differentially expressed genes in ciliated cells from healthy and IPF tissue. (**A**) Heatmap showing differentially expressed genes in the clusters (0–6; see Figure 2D) identified from the sequencing analysis. (**B**) UMAP plots showing the expression of 3 top upregulated genes in healthy ciliated cells versus IPF ciliated cells. (**C**) UMAP plots showing the expression of 3 top upregulated genes in IPF ciliated cells versus healthy ciliated cells. IPF *n* = 2, healthy *n* = 4.

The initial cluster analysis revealed that the majority of the primary IPF cells clustered separately from the primary healthy epithelial cells, demonstrating a difference in gene expression between the healthy and diseased samples (Figure 2C). Overall, the cells were divided into seven clusters, and by using the expression of known epithelial cell type markers, we could confirm that the majority of sequenced cells were airway epithelial cells (Figure 2D,E). Interestingly, three separate clusters all consisted of ciliated cells (FOXJ1^+^); however, sample identity indicates that the ciliated cells in cluster 2 were all from IPF patients, while clusters 1 and 5 contained healthy ciliated cells, demonstrating that ciliated cells in IPF differed in gene expression from their healthy counterparts. This can also be seen in Figure 3A, which shows a heatmap that illustrates the expression of differentially expressed genes in the seven clusters. Taken together, this indicates that ciliated cells are molecularly altered in IPF.

Next, we identified the top differentially expressed genes in the IPF ciliated cell cluster in comparison to the clusters of healthy ciliated cells. The most differentially expressed genes of interest were selected by sorting the genes expressed in clusters 1 and 5 (healthy ciliated cells) and cluster 2 (IPF ciliated cells) by the lowest *p*-value followed by the highest fold change in gene expression. Figure 3B shows the top three upregulated genes of interest in healthy ciliated cells vs. IPF ciliated cells: FTL, GSTA2 and FOS. Correspondingly, Figure 3C shows three genes of interest that were highly upregulated in IPF ciliated cells: SYT8, FTO and NEAT1. FTL encodes the light chain of Ferritin, a protein consisting of several subunits that is responsible for intracellular iron storage as well as transport of excess iron [13]. Interestingly, excess iron accumulation in the lung has been associated with IPF and has been shown to contribute to IPF pathogenesis [14]. SYT8 is a regulator of exocytosis, which in turn can influence the microenvironment of the lung and lead to disease development [15]. FTO and NEAT1, though not previously characterized in IPF, have both been implicated in promoting various forms of lung cancer [16,17,18].

### 3.3. Ciliated Cells in IPF Lung Epithelium Exhibit Increased Expression of Ciliated Pathways

Next, we performed gene ontology analysis of all the differentially expressed genes in IPF ciliated cells versus healthy control ciliated cells. Table 1 shows the significantly upregulated biological processes in healthy ciliated cells together with the specific genes involved in each process. Correspondingly, the same analysis is shown for IPF ciliated cells in Table 2. For healthy ciliated cells, general biological processes seemed to be maintained, such as RNA processing, translation, mitochondrial electron transport and epithelial cell differentiation. However, in ciliated cells from IPF patients, a large part of significantly upregulated pathways were directly linked to cilium morphogenesis, which is in line with previous findings showing increased cilia in IPF [19].

### 3.4. Ciliated Cells in IPF Patients Display Reduced FTL Protein Levels

**Figure 4:**
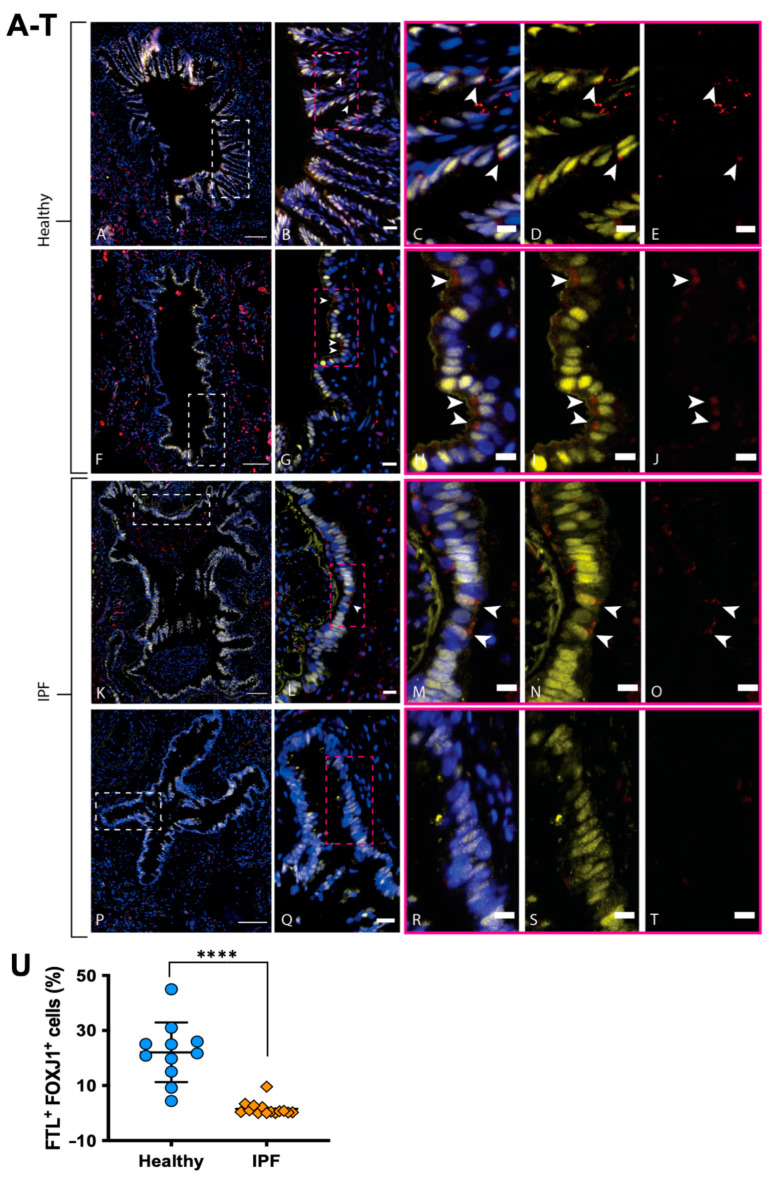
FTL expression in the airway epithelium of healthy and IPF distal lung tissue. Healthy bronchioles (**A**,**F**) with pseudostratified epithelium depicts FTL staining (red) in apical lining of ciliated epithelial cells (FOXJ1, yellow) (DAPI, blue). (**B**,**G**) show enlarged area (white box) from (**A**) and (**F**), respectively. (**C**–**E**) and (**H**–**J**) show enlarged area (pink box) from (**B**,**G**), respectively, with arrows indicating FTL^+^ ciliated cells (FOXJ1^+^). IPF bronchiole (**P**) shows epithelium devoid of FTL staining (**Q**–**T**), while IPF bronchiole (**K**) shows positive staining of FTL in pseudostratified epithelium with apical localization (**L**–**O**, arrows). IPF bronchiole (**K**) also shows signs of ongoing inflammation with macrophages present in luminal space, highly expressing FTL. (**U**): Number of FTL^+^ ciliated cells in healthy and IPF airways, expressed as the percentage of cells among the total number of FOXJ1^+^ cells in each analyzed airway. Images are representative of examined lung tissue samples. IPF *n* = 2 individuals for pictures, *n* = 4 for quantification, healthy *n* = 2 for pictures, *n* = 4 for quantification. Scale bars: 100 µm (**A**,**F**,**K**,**P**), 20 µm (**B**,**G**,**L**,**Q**), 10 µm (**C**–**E**,**H**–**J**,**M**–**O**,**R**–**T**). **** *p* < 0.0001.

Among the genes of interest that were differentially expressed in ciliated cells, FTL was upregulated in healthy individuals in comparison to IPF patients. Since iron accumulation in IPF lung tissue has been implicated in pathological progression [20] and is partly controlled by FTL [13], healthy and IPF tissue sections were stained for FTL together with FOXJ1 in order to verify our single-cell RNA analysis at the protein level, since FTL is known to be post-transcriptionally regulated [21]. In line with the RNA sequencing, antibody labeling of distal lung tissue detected an increase in cells costained for FTL (red) and FOXJ1 (yellow) in healthy airways in comparison to IPF airways (Figure 4). FTL was found to be expressed in ciliated airways of healthy distal lung tissue (Figure 4A–J), seen at the apical end of FOXJ1^+^ ciliated cells (Figure 4C–E,H–J). In contrast, in IPF ciliated airways, the FTL protein expression was more sporadic (Figure 4K–O), and in some airways very little expression was found (Figure 4P–T). Nevertheless, in some IPF airways, we found signs of ongoing inflammation with FTL-expressing macrophages (Figure 4K). This is in line with other studies showing strong FTL expression in macrophages [22], and validates our staining assays.

In order to assess the level of FTL protein expression in ciliated cells specifically, we next used Qupath to quantify FTL in multiple ciliated airways and patients (Figure 4U), demonstrating a significant decrease in FTL+ ciliated cells in airways from IPF patients compared to healthy individuals; the difference in mean percentage of FTL + FOXJ1^+^ cells (± SD) was 20.54 ± 2.980% (*p* value < 0.0001).

## 4. Discussion

Previously, single-cell RNA sequencing studies have been performed on IPF tissue, where disease-specific changes in gene expression were observed in alveolar and basal cells [23,24]. However, IPF lungs have previously been proven to contain several types of nontypical epithelial structures, such as honeycomb cysts and (less commonly) bronchiolization, where ciliated cells are prominent [5,6]. In addition, cilia may be responsible for signaling between cells for immune cell recruitment or fibroblast migration, possibly contributing to the pathogenesis [9]. Therefore, the aim of this study was to examine and compare ciliated cells in healthy and IPF lung tissue, in order to elucidate their possible role in disease development and to explore future targets for cell therapy.

The gene ontology analysis suggested pathways connected to cilium formation and movement were upregulated in IPF ciliated cells, indicating an active role of ciliated cells in the diseased tissue, which was of interest for further study. This is in line with previous findings demonstrating an upregulation of cilia in all IPF airway cells [19].

In examining the genes with the highest fold change in expression between ciliated cells from healthy and IPF tissue, IPF ciliated cells were found to highly express FTO and NEAT1, both markers implicated in lung cancer development. In addition, SYT8 was highly expressed in IPF ciliated cells, suggesting an active formation of extracellular vesicles that may propagate the disease by influencing the cellular microenvironment.

FTL was among the top upregulated genes by healthy cells (and therefore comparatively downregulated in IPF). In line with this finding, FTL protein expression was found in a lower percentage of airway ciliated cells in IPF tissue sections compared to healthy airways, verifying the sequencing data. Iron is stored intracellularly and metabolized by ferritin, a protein consisting of the ferritin light chain (FTL) and the ferritin heavy chain (FTH1) [25]. FTL is responsible for protein stability and iron nucleation, while FTH1 oxidates ferrous ions, thus protecting the cellular environment from radical formation [13]. Apart from the harmful formation of free radicals, excess iron in the airways may also be beneficial to the proliferation of pathogens during lung infections [26]. In addition, iron can be inhaled via pollution or cigarette smoke, and is thus especially prone to have a negative effect on lung disease development [27]. It has also been shown that iron accumulation and overload in the lung is detrimental to lung function and often present in patients with IPF [20]. Following up on previous findings, in 2020, Ali et al. [14] used iron chelators to prevent a decline in lung function in IPF patients, suggesting that free iron in the lung negatively affects the outcome of IPF pathogenesis and is a possible target for future IPF treatment.

Thus, further elucidating the role of iron in chronic and interstitial lung diseases such as IPF is important and of interest in this study. A possible effect of the lower levels of FTL expressed by the IPF ciliated cells may be that free iron is not stored and protected from oxidation inside the cells and instead accumulates in the airways, which can influence the environment and be damaging to the epithelium. Furthermore, the lack of FTL-mediated storage of iron in the cells may inhibit regular metabolic processes. Therefore, future studies should investigate the importance of ferritin levels in IPF in order to address the possibility of targeting this as a treatment or amelioration of the disease symptoms. For this, it would be advantageous to include more cases to strengthen the results, and especially to include more functional assays. Additionally, it would be of interest to examine other chronic fibrotic lung diseases, e.g., fibrotic hypersensitivity pneumonitis, in which honeycombing may exist and bronchiolization is more common than in IPF.

Still, these findings may have implications on future cell-based therapies attempting to regenerate damaged lung tissue in an otherwise unchanged disease microenvironment. Even though new airway stem cells (such as basal cells) could be transplanted, the differentiated progeny may still become affected by factors such as extracellular iron levels and ciliated cell-mediated signaling, possibly reversing any therapeutic effects and maintaining the disease phenotype. For example, if the reduced levels of FTL still persist after successful transplantation of healthy stem cells, ciliated cells lacking FTL could potentially go through an epithelial–mesenchymal transition [28] and add to fibrosis, thus continuing to fuel the disease. Moreover, whether the downregulation of FTL in IPF ciliated cells is due to a change postdifferentiation or if this shift originates in the basal cell(s) that give rise to the ciliated cells still needs to be determined. In either case, the cause of the downregulation should be further studied to resolve its role in the disease, and other neighboring cell types should be assessed for FTL expression as well. Taken together, this argues for the need of complementary treatments in order to develop successful cell-based therapies and stimulate healthy differentiation.

In conclusion, this study showed a differential gene expression between ciliated cells from healthy and IPF distal lung tissue, which is also reflected at the protein level. Ciliated cells make up a large portion of airways and have important tasks in normal lung function, such as protection from pathogens and mediation of cell signaling. Therefore, it is important to further examine these findings in order to understand the possible effects of these aberrant ciliated cells and what genes or pathways may contribute to IPF pathogenesis. FTL was shown to be under-expressed in ciliated cells from IPF tissue, which suggests that a disease-related association with iron storage may contribute to a decline in lung function and possibly constitutes a target for development of future IPF therapies.

## Figures and Tables

**Table 1:** Pathways upregulated in healthy ciliated cells.

GO Term	*p* Value	Genes
GO:0006614~SRP-dependent cotranslational protein targeting to membrane	1.56 × 10^−18^	RPL30, RPS8, RPLP1, RPL34, RPS6, RPL11, SRP54, RPS3A, RPL10A, RPS4X, RPS14, RPS15A, RPS19, RPL13, RPL29, RPS27A, RPS11, RPL28, RPL39, RPS24, RPS13, RPL19
GO:0006413~translational initiation	3.00 × 10^−17^	RPL30, RPS8, RPLP1, RPL34, RPS6, RPL11, RPS3A, RPL10A, EIF1, RPS4X, RPS14, RPS15A, RPS19, RPL13, RPL29, RPS27A, RPS11, RPL28, EIF3D, RPL39, RPS24, EIF3A, RPS13, RPL19
GO:0000184~nuclear-transcribed mRNA catabolic process, nonsense-mediated decay	2.60 × 10^−16^	RPL30, RPS8, RPLP1, RPL34, RPS6, RPL11, RPS3A, RPL10A, RPS4X, RPS14, RPS15A, RPS19, MAGOH, RPL13, RPL29, RPS27A, RPS11, RPL28, RPL39, RPS24, RPS13, RPL19
GO:0019083~viral transcription	1.18 × 10^−15^	RPL30, RPS8, RPLP1, RPL34, RPS6, RPL11, RPS3A, RPL10A, RPS4X, RPS14, RPS15A, RPS19, RPL13, RPL29, RPS27A, RPS11, RPL28, RPL39, RPS24, RPS13, RPL19
GO:0006364~rRNA processing	6.37 × 10^−13^	RPL30, RPS8, RPLP1, RPL34, RPS6, RPL11, RPS3A, RPL10A, RPS4X, RPS14, EBNA1BP2, RPS15A, RPS19, RPL13, PIH1D2, RPL29, RPS27A, RPS11, RPL28, RPL39, RPS24, RPP38, RPS13, RPL19
GO:0006412~translation	3.00 × 10^−12^	RPL30, RPL34, RPLP1, RPL11, RRBP1, RPL10A, MRPL41, RPS4X, RPS14, RPS15A, RPS19, RPL13, RPS27A, RPS11, RPL39, RPS13, RPL19, RPS8, RPS6, RPS3A, MRPL23, RPL29, RPL28, SLC25A11, RPS24
GO:0043488~regulation of mRNA stability	6.20 × 10^−6^	YTHDF2, SET, PSMA4, PSMA1, PSMD13, APEX1, PSMD2, UBC, PSMD3, RPS27A, YWHAZ
GO:0060071~Wnt signaling pathway, planar cell polarity pathway	1.17 × 10^−4^	PSMA4, PSMA1, PSMD13, PSMD2, UBC, PSMD3, PFN1, RPS27A, AP2M1
GO:0098869~cellular oxidant detoxification	1.30 × 10^−4^	GSTK1, PRDX5, GPX4, GSTP1, GSTA1, CAT, TXN, TXNDC17
GO:0006457~protein folding	1.63 × 10^−4^	CCT3, DNAJA1, FKBP1A, LMAN1, HSP90AA1, ST13, RUVBL2, MLEC, TXN, CCT7, PDIA6, PFDN5
GO:0000398~mRNA splicing, via spliceosome	2.61 × 10^−4^	PRPF40A, LSM7, HNRNPK, SNRPD2, PCBP1, MAGOH, SRSF2, POLR2F, SRSF3, SNRPA1, POLR2H, HNRNPA1, SRSF7
GO:0000302~response to reactive oxygen species	3.81 × 10^−4^	PRDX5, GSTP1, PRDX1, CAT, P4HB, TXN
GO:0098609~cell-cell adhesion	4.62 × 10^−4^	DDX3X, ANXA2, RPL34, HDLBP, YWHAZ, CORO1B, MPP7, PCMT1, HNRNPK, PCBP1, PRDX1, PFN1, RPL29, S100A11
GO:0002479~antigen processing and presentation of exogenous peptide antigen via MHC class I, TAP-dependent	5.15 × 10^−4^	PSMA4, PSMA1, PSMD13, PSMD2, PSMD3, HLA-B, B2M
GO:0038061~NIK/NF-kappaB signaling	6.62 × 10^−4^	PSMA4, PSMA1, PSMD13, PSMD2, UBC, PSMD3, RPS27A
GO:0051436~negative regulation of ubiquitin-protein ligase activity involved in mitotic cell cycle	9.76 × 10^−4^	PSMA4, PSMA1, PSMD13, PSMD2, UBC, PSMD3, RPS27A
GO:0006120~mitochondrial electron transport, NADH to ubiquinone	0.001108056	NDUFS8, NDUFS7, NDUFA5, NDUFA4, NDUFB11, NDUFS3
GO:0050434~positive regulation of viral transcription	0.001131143	POLR2F, RSF1, POLR2H, PFN1, NELFE
GO:0090090~negative regulation of canonical Wnt signaling pathway	0.001306836	SOX2, PSMA4, PSMA1, PSMD13, PSMD2, UBC, PSMD3, IGFBP2, RPS27A, PFDN5
GO:0051437~positive regulation of ubiquitin-protein ligase activity involved in regulation of mitotic cell cycle transition	0.001394273	PSMA4, PSMA1, PSMD13, PSMD2, UBC, PSMD3, RPS27A
GO:0043161~proteasome-mediated ubiquitin-dependent protein catabolic process	0.00169636	PCNP, PPP2CB, PSMA4, PSMA1, PSMD13, PSMD2, UBC, UBE2D3, PSMD3, FBXO15, RPS27A
GO:0031145~anaphase-promoting complex-dependent catabolic process	0.001703314	PSMA4, PSMA1, PSMD13, PSMD2, UBC, PSMD3, RPS27A
GO:0009060~aerobic respiration	0.001853146	CHCHD5, SURF1, PANK2, CAT, UQCRC2
GO:0033209~tumor necrosis factor-mediated signaling pathway	0.002977448	PSMA4, PSMA1, PSMD13, PSMD2, UBC, PSMD3, RPS27A, TXNDC17
GO:0090263~positive regulation of canonical Wnt signaling pathway	0.003270083	PSMA4, PSMA1, PSMD13, PSMD2, UBC, PSMD3, PIN1, RPS27A
GO:1901687~glutathione derivative biosynthetic process	0.005049175	GSTK1, GSTP1, GSTA2, GSTA1
GO:0030855~epithelial cell differentiation	0.005354618	GSTK1, LGALS3, GSTA2, GSTA1, PGK1, ANXA7
GO:0006283~transcription-coupled nucleotide-excision repair	0.006767992	COPS6, UBC, TCEA1, POLR2F, POLR2H, RPS27A
GO:0002223~stimulatory C-type lectin receptor signaling pathway	0.006986379	PSMA4, PSMA1, PSMD13, PSMD2, UBC, PSMD3, RPS27A
GO:0043066~negative regulation of apoptotic process	0.007011217	NPM1, ANXA1, DDX3X, GSTP1, RPS6, RPS3A, YWHAZ, DNAJA1, PRDX5, HNRNPK, CAT, UBC, TAX1BP1, RPS27A, SQSTM1, TPT1
GO:0010467~gene expression	0.007303284	HNRNPK, PCBP1, POLR2F, POLR2H, HNRNPA1
GO:0016236~macroautophagy	0.007564389	MAP1LC3B, MAP1LC3A, UBC, RPS27A, DYNLL1, SQSTM1
GO:0045454~cell redox homeostasis	0.007986077	PRDX5, APEX1, PRDX1, P4HB, TXN, PDIA6
GO:0038095~Fc-epsilon receptor signaling pathway	0.008269841	PSMA4, PSMA1, PSMD13, PSMD2, UBC, PSMD3, FOS, CALM1, RPS27A
GO:0006521~regulation of cellular amino acid metabolic process	0.009035625	PSMA4, PSMA1, PSMD13, PSMD2, PSMD3
GO:0006511~ubiquitin-dependent protein catabolic process	0.009376141	PSMA4, PSMA1, PSMD13, USP10, ADRM1, UBE2D3, PSMD3, SQSTM1, HERPUD1
GO:0042493~response to drug	0.010386588	TGIF1, HSP90AA1, ABCD3, ANXA1, XRCC5, APEX1, IGFBP2, CAT, SRP54, GNAS, FOS, B2M
GO:0006405~RNA export from nucleus	0.011733373	MAGOH, SRSF2, SRSF3, HNRNPA1, SRSF7
GO:0032480~negative regulation of type I interferon production	0.012113648	UBC, TAX1BP1, PIN1, RPS27A
GO:0006368~transcription elongation from RNA polymerase II promoter	0.012537861	CCNK, ADRM1, TCEA1, POLR2F, POLR2H, NELFE
GO:0000462~maturation of SSU-rRNA from tricistronic rRNA transcript (SSU-rRNA, 5.8S rRNA, LSU-rRNA)	0.01445558	RPS14, RPS19, RPS8, RPS24

**Table 2:** Pathways upregulated in IPF ciliated cells.

GO Term	*p* Value	Genes
GO:0060271~cilium morphogenesis	4.02 × 10^−24^	CEP126, TTC26, IFT172, CEP164, CCDC28B, RPGR, TCTN2, IQUB, DZIP1L, TEKT3, CEP290, BBS5, DYNC2H1, IFT140, DNAAF1, RFX2, TTC21A, RFX3, IFT80, RPGRIP1L, WDR35, KIF27, DNM2, IFT88, CFAP221, TMEM138, WDPCP, CFAP54, INTU, ARL6, DNAH5, TTBK2, STK36, TMEM67, TTC21B, NPHP3, BBS1, GSN, WDR19, IFT122, FOXJ1, FUZ, TMEM231, AHI1, TMEM17, TTLL3, MKS1, CEP162, DZIP1
GO:0042384~cilium assembly	2.49 × 10^−22^	LAMA5, CEP126, TTC26, INTU, ARL6, DNAH5, IFT172, CEP164, CCDC28B, TTBK2, RPGR, STK36, TMEM67, TCTN2, DZIP1L, BBS9, BBS5, CEP290, DYNC2H1, BBS1, DNAI2, IFT140, WDR19, RAB3IP, RFX2, IFT122, RFX3, FOXJ1, FUZ, IFT80, RPGRIP1L, WDR35, KIF27, TMEM231, AHI1, TMEM17, TTLL3, TMEM138, ALMS1, MKS1, CEP162, WDPCP, CFAP54, DZIP1, CLUAP1
GO:0003341~cilium movement	6.77 × 10^−12^	CCDC39, RSPH4A, DNAH11, DNAH1, DNAI2, CFAP73, DNAH7, DNAAF1, DNAH5, CCDC114, CFAP100, CFAP221, HYDIN, DNAI1, CCDC151, CCDC40
GO:0008380~RNA splicing	4.28 × 10^−7^	RBM25, DDX23, AKAP8L, RNPC3, CCAR2, IWS1, SNRNP70, ZNF326, RBM5, SRSF11, RBM10, RBM38, RBM39, MBNL1, ZRANB2, SCAF11, PRPF4B, THOC2, SCAF1, SON, ZNF638, PRPF3, LUC7L3, RBM20, SRSF4, SREK1, CDK12, CLASRP, TARDBP, SUGP2
GO:0060285~cilium-dependent cell motility	9.68 × 10^−7^	DNAH3, CCDC39, DNAH1, DNAH2, DNAH7, DNAAF2, RFX3, CFAP44
GO:0036159~inner dynein arm assembly	4.50 × 10^−6^	CCDC39, CFAP100, DNAH1, CFAP73, DNAH7, DNAAF1, TEKT2, CCDC40
GO:0061512~protein localization to cilium	5.66 × 10^−6^	BBS1, ARL6, IFT140, TTC21B, IFT122, TTC21A, BBS9, CSNK1D, WDR35
GO:0035721~intraciliary retrograde transport	9.95 × 10^−6^	DYNC2H1, IFT140, TTC21B, WDR19, IFT122, TTC21A, WDR35
GO:0035058~nonmotile primary cilium assembly	2.37 × 10^−5^	BBS1, CEP126, TMEM17, C2CD3, CCDC13, MKS1, FUZ, IFT80, CSNK1D, PIBF1
GO:0035082~axoneme assembly	4.09 × 10^−5^	RSPH4A, RP1, CFAP74, LRGUK, SPEF2, TTLL3, CFAP46, CLUAP1
GO:0043484~regulation of RNA splicing	4.52 × 10^−5^	RBM38, CLK1, MBNL1, SON, SNRNP70, CELF1, HNRNPH1, ZNF326, CDK12, CLK4
GO:0006397~mRNA processing	5.01 × 10^−5^	RBM25, CELF1, SRSF1, AKAP8L, CCAR2, IWS1, U2AF2, SNRNP70, ZNF326, SRSF11, RBM10, RBM38, RBM39, MBNL1, ZRANB2, SCAF11, SCAF1, SON, PRPF3, RBM20, SRSF4, SREK1, CDK12, CLASRP, TARDBP, RBM23, SUGP2
GO:0001843~neural tube closure	7.16 × 10^−5^	SDC4, IFT172, SEMA4C, COBL, TSC2, SHROOM3, IFT122, TULP3, FUZ, ARID1A, CELSR1, SPINT1, MKS1, PLXNB2, CLUAP1, PHACTR4
GO:0006468~protein phosphorylation	7.71 × 10^−5^	ULK4, IKBKB, NRBP2, AKAP13, PPP4R1, CDK20, TLK1, NEK3, MAP3K5, STRADA, DAPK1, PRKCD, DYRK1B, CSNK1D, PRPF4B, CSNK1E, PASK, GAK, MAK, SIK3, BIRC6, GAS6, ALPK1, CSNK1G2, CDKL1, CAMK2D, LTK, ATP23, NPR2, PRKCZ, STK3, STK36, PHKG2, ERBB2, STK38, MAP4K4, DMPK, NEK5, CDC42BPG, MOK, BRAF, HIPK1, HIPK3, MAPK10, FER, WNK1, NEK10, AAK1, CDK10, COQ8B, PKN1
GO:0007368~determination of left/right symmetry	1.20 × 10^−4^	DYNC2H1, DNAH11, DNAI2, ARL6, IFT140, DNAH5, FOXJ1, RPGRIP1L, PCSK5, MKS1, NPHP3, DNAI1, CCDC151
GO:0044458~motile cilium assembly	1.81 × 10^−4^	CCDC39, DNAAF3, DNAAF1, DMD, BBOF1, BBS5, CCDC40
GO:0070286~axonemal dynein complex assembly	1.83 × 10^−4^	CCDC39, DNAAF3, DNAAF2, DNAAF1, CCDC151, CCDC40
GO:0098609~cell-cell adhesion	1.87 × 10^−4^	MACF1, PDXDC1, ARHGAP18, CLINT1, BAIAP2L1, MPRIP, PPME1, TRIM29, STK38, MYO6, PACSIN2, EPS8L1, FLNB, EPS8L2, ERC1, LRRFIP1, ARGLU1, SPTBN1, PAK4, SH3GLB2, LYPLA2, IST1, CSNK1D, BAIAP2, RAB11B, HCFC1, ATXN2L, AFDN, EXOC3, ESYT2, TJP2, PLEC, EPHA2, EIF4G1
GO:0051056~regulation of small GTPase mediated signal transduction	2.47 × 10^−4^	ARHGEF12, GDI1, RALGAPA1, RALGAPA2, ARHGEF17, FAM13A, ARAP2, TSC2, ARHGAP18, ARHGAP39, MYO9A, SIPA1L3, ARHGAP24, AKAP13, ABR, RHOT2, RALGAPB, ARHGEF4, SRGAP3, ARHGEF2, SRGAP2
GO:0007224~smoothened signaling pathway	3.03 × 10^−4^	TTC26, IFT172, IFT80, HIPK1, TTBK2, TMEM231, HHAT, TMEM17, TTC21B, TCTN2, IQUB, WDPCP, DZIP1, CLUAP1
GO:0043547~positive regulation of GTPase activity	4.22 × 10^−4^	GDI1, FAM13A, ARHGAP39, SIPA1L3, RPGR, FGF5, AKAP13, HERC2, SYNGAP1, DNM1L, BCAS3, DENND2C, ARHGEF12, ARHGEF17, ELMOD1, ARAP2, TSC2, AGAP9, RASA3, TBC1D20, NRG4, AKAP9, SPATA13, RAPGEF1, ARHGEF4, PKP4, DENND6B, ARHGEF2, ECT2L, LLGL2, TBC1D19, CAMK2D, ARHGAP18, AGAP4, ABR, ALS2CL, ERBB4, ERBB2, EPS8L1, EPS8L2, SRGAP3, SRGAP2, RALGDS, CYTH1, SPTBN1, GIT2, RAB3IP, ARHGAP27, MYO9A, ARHGAP24, PTK2, AFDN, DLG4, ST5, SGSM2, PLXNB1, AGRN
GO:0007030~Golgi organization	6.13 × 10^−4^	DYNC2H1, BCAS3, COG7, CSNK1D, SYNE1, VMP1, GAK, TBC1D20, GORASP1, GOLGB1, TRIP11, KIFC3, CLASP1, CLASP2
GO:0018105~peptidyl-serine phosphorylation	7.64 × 10^−4^	SMG1, CAMK2D, DMPK, PRKCD, CSNK1D, CSNK1E, PRKCZ, PKD1, HIPK3, TTBK2, CLK1, GRK2, AKT2, STK38, RICTOR, PKN1, GAS6, CSNK1G2, ATR
GO:0008589~regulation of smoothened signaling pathway	7.89 × 10^−4^	INTU, C2CD3, ARL6, IFT140, TTC21B, FUZ, RPGRIP1L
GO:0003351~epithelial cilium movement	7.98 × 10^−4^	SPAG17, DNAH1, STK36, DNAI1, KIF27, CCDC40
GO:0060287~epithelial cilium movement involved in determination of left/right asymmetry	8.68 × 10^−4^	CCDC39, DNAAF1, NPHP3, RFX3, CCDC40
GO:0016337~single organismal cell-cell adhesion	0.001486135	VEZT, PTPRU, ADGRV1, CTNND1, ICAM2, ICAM5, PKD1, VMP1, ARVCF, DLG1, CDH1, NPHP1, RAPGEF1, FAT1, CTNNB1, PKP4
GO:0036158~outer dynein arm assembly	0.001676592	DNAI2, CCDC114, DNAAF1, DNAH5, DNAI1, CCDC151
GO:0007163~establishment or maintenance of cell polarity	0.001694226	DLG1, PARD3, FAT1, NPHP3, RPGRIP1L, CLASP1, SYNE2, CLASP2
GO:0003356~regulation of cilium beat frequency	0.002215945	CCDC39, DNAH11, DNAAF1, CCDC40
GO:0035469~determination of pancreatic left/right asymmetry	0.002215945	CCDC39, DNAAF1, NPHP3, CCDC40
GO:0006351~transcription, DNA-templated	0.002281724	HDAC10, WWC1, CTNND1, ATN1, ZBTB20, CCAR2, CCAR1, ZNF83, ZNF606, ZMIZ2, ZNF84, CPNE1, ZNF326, CCNL2, KMT5A, ZNF446, SOX6, ZNF444, TRIM22, DDX17, ZNF440, ZNF19, KMT5C, LMO3, ZNF160, RFX2, PTOV1, RFX3, RFX1, EMSY, SUPT7L, FOXP1, ZNF91, DMTF1, TBL1XR1, MAK, ZNF439, MZF1, SF1, ZNF431, ZNF395, CASZ1, INO80E, CTBP2, ZNF23, PHF21A, NPAS2, SBNO2, ZNF708, ATXN1, NKX2-1, TP53BP1, ARGLU1, GTF2IRD2B, ZNF664, BANP, ZSCAN18, SMAD3, GTF2IRD2, ZBTB16, NR2F1, CNOT10, FOXN3, MED13L, MOV10, GON4L, BCL6, CNOT1, MAFK, MMS19, CNOT9, ZNF254, CREBZF, PHF3, DIDO1, ZNF253, KDM5C, ZNF493, CHD9, CHD8, CDCA7L, CHD7, AKAP8L, ZNF44, EFCAB6, CHD2, ZNF280D, SIN3A, CHMP1A, ZNF644, LRRFIP1, ZNF763, PELP1, NCOA2, BCAS3, RBM14, PAWR, IL16, ARID1A, ZFP90, SREBF2, NCOR2, ELF2, ZNF638, CRY2, CRY1, BDP1, GTF3C1, GTF3C3, KDM3B, ATF6B, SFSWAP, SATB1, CXXC1, ZNF518A, ZBTB44, ZNF69, IWS1, HDAC7, RXRA, MTA1, ERBB4, POLR2B, FAM120B, ZNF506, ERBB2, TP53INP1, E2F3, STAT6, APBB1, ZNF621, ZNF586, E2F8, HES4, MLXIP, SPEN, RBM39, KDM4B, EYA1, PCGF3, SAMD4B, ZNF76, HIPK1, YY1AP1, ZNF33B, NFIB, CTNNB1, ZNF611, PKN1, LPIN1, PAXBP1, SSBP3, MPHOSPH8
GO:0016477~cell migration	0.002504521	LAMA5, SDC4, TNK2, WWC1, USP33, SORBS2, DGKZ, PRKCZ, PTPRF, ABI2, ERBB4, PEAK1, TAOK2, SPATA13, FAT1, ABL1, PIP5K1A, PLXNB1, ELMO3, GAS6, EPHA2, PAK4
GO:0046777~protein autophosphorylation	0.002504521	DDR1, SMG1, CAMK2D, DAPK1, PASK, CLK4, MAPK15, PTK2, IGF1R, CLK1, FER, STK33, WNK1, ERBB4, PEAK1, MAK, ERBB2, AAK1, ABL1, CDK12, CSNK1G2, ATR
GO:0006897~endocytosis	0.002600443	TNK2, USP33, INPPL1, TSC2, AP2A1, CSNK1D, CSNK1E, CLINT1, C9ORF72, DNM2, MYO6, AAK1, ANKFY1, ESYT2, DNM1L, RAB5A, ATP9B, CSNK1G2, BCL2L1
GO:0001701~in utero embryonic development	0.003188547	SLC34A2, MBNL1, SMAD3, C2CD3, PRKCSH, ANKRD11, CHD8, WDR19, CHD7, SRSF1, RPGRIP1L, PKD1, FOXP1, MUC1, RXRA, C6, SCO2, SIN3A, MYH9, CTNNB1, SOX6, BCL2L1, WDTC1
GO:0010506~regulation of autophagy	0.003466465	VMP1, DAPK1, USP33, TP53INP1, PSAP, ABL1, ITPR1, EP300, HSPB1, FBXL2
GO:0071910~determination of liver left/right asymmetry	0.004225947	CCDC39, DNAAF1, NPHP3, CCDC40
GO:0060294~cilium movement involved in cell motility	0.004225947	DNAH1, CFAP46, CFAP54, GAS8
GO:0007018~microtubule-based movement	0.004348348	DYNC2H1, DNAH3, DNAH12, DNAH2, DNAH10, DNAH5, DNAH6, DNHD1, AP2A1, KIF27, KIF19, KIF13A, KIFC3
GO:0043001~Golgi to plasma membrane protein transport	0.005604612	BBS1, MACF1, RAB31, KIF13A, GCC2, ANK3, SPTBN1
GO:0043984~histone H4-K16 acetylation	0.006667143	KMT2A, KANSL1, KANSL1L, OGT, MSL1, HCFC1
GO:0034453~microtubule anchoring	0.006890861	CCDC187, FGFR1OP, GCC2, CLASP1, CLASP2
GO:0032956~regulation of actin cytoskeleton organization	0.008098045	LRP1, PRKCD, ABL1, ARHGAP18, RICTOR, CELSR1, BAIAP2, GPM6B, CLASP2
GO:0021591~ventricular system development	0.00917602	TTC21B, MBOAT7, HYDIN, AK8, KIF27
GO:0090630~activation of GTPase activity	0.009840864	TBC1D8B, BCAS3, RALGAPB, RALGAPA1, AKT2, RALGAPA2, SGSM3, SGSM2, TBC1D8, PIP5K1A, FOXJ1, EPHA2
GO:0051301~cell division	0.0122476	ANKLE2, NUMA1, CDCA7L, CEP164, KATNB1, PMF1, POGZ, CNTRL, CHMP1A, CDK20, ZNF207, NEK3, MAP4, KMT5A, CLASP1, CLASP2, DIS3L2, LMLN, LIG1, IST1, SPICE1, ATAD3B, CHFR, CCNA1, MAU2, STAG2, PPP2R2D, CDK10, ANAPC4, MAPRE3, BIRC6, ARHGEF2, CDK13, LLGL2
GO:0000226~microtubule cytoskeleton organization	0.012507657	FER, SON, DST, RNF19A, ULK4, TACC2, TTL, PRKCZ, PTK2, CLASP1, CLASP2
GO:0001947~heart looping	0.013018715	CCDC39, AHI1, SMAD3, C2CD3, IFT172, DNAAF1, NPHP3, CLUAP1, BBS5, CCDC40
GO:0045880~positive regulation of smoothened signaling pathway	0.014858592	DYNC2H1, POR, INTU, STK36, IFT172, IFT80
GO:0030010~establishment of cell polarity	0.014858592	WWC1, RICTOR, NEK3, PRKCZ, PKD1, PTK2

## Data Availability

All data are in the process of being uploaded to the National Center for Biotechnology Information (Gene Expression Omnibus).

## Supplementary Materials

The following supporting information can be downloaded at: https://www.mdpi.com/article/10.3390/cells11061031/s1, Table S1: Lung donor information, Table S2: Antibodies used in FACS experiments, Table S3: Antibodies used in immunofluorescence staining of human lung tissue.
