# Supplementary material for: Ciliated (FOXJ1+) Cells Display Reduced Ferritin Light Chain in the Airways of Idiopathic Pulmonary Fibrosis Patients
Source: Cells. 2022 Mar 18;11(6):1031. doi: 10.3390/cells11061031 (PMC8947470; doi:10.3390/cells11061031)
Supplement: Supplementary file 1 [file cells-11-01031-s001.zip › cells-1584808-supplementary.pdf]

**Supplementary table S1.** Lung donor information.

| Patient ID | Age | Disease | Smoker | Sex | Used in experiments |
|---|---|---|---|---|---|
| #1 | 65 | IPF | Former | Female | IHC (Fig. 4) |
| #2 | 62 | IPF | Former | Female | IHC (Fig. 1) |
| #3 | 67 | IPF | Never | Male | RNA Sequencing, IHC (Fig. 4) |
| #4 | 57 | IPF | Former | Male | Sequencing, IHC (not pictured) |
| #5 | 62 | Healthy | Never | Male | IHC (Fig. 1, Fig. 4) |
| #6 | 68 | Healthy | Never | Male | IHC (Fig. 1, Fig. 4) |
| #7 | 65 | Healthy | Former | Male | RNA Sequencing, IHC (not pictured) |
| #8 | 47 | Healthy | Never | Male | RNA Sequencing |
| #9 | 31 | Healthy | Never | Male | RNA Sequencing |
| #10 | 23 | Healthy | Never | Male | RNA Sequencing |

**Supplementary table S2.** Antibodies used in FACS experiments.

| Antibody | Supplier | Catalog Number | Dilution |
|---|---|---|---|
| 7AAD | Sigma | A9400-5MG | 1/100 |
| CD45 - APC | Biolegend | 304010 | 1/50 |
| CD31 – APCeFluor780 | Invitrogen | 15599536 | 1/50 |
| NGFR - FITC | Biolegend | 345104 | 1/50 |

**Supplementary table S3.** Antibodies used in immunofluorescence staining of human lung tissue.

| Antigen | Supplier | Catalog Number | Dilution | Secondary antibody | Supplier | Catalog Number | Dilution |
|---|---|---|---|---|---|---|---|
| KRT5 | Cell signaling technology | 258075 | 1/100 | AF647 | Abcam | A-21246 | 1/200 |
| FOXJ1 | Invitrogen | 14-9965-82 | 1/100 | AF555 | Invitrogen | A-21127 | 1/200 |
| FTL | Invitrogen | PA5-83567 | 1/200 | AF647 | Abcam | A-21246 | 1/200 |
